# Prevalence of Severe Disability and Its Associated Factors in Northwestern Ethiopia: Evidence From Dabat District of Amhara National Regional State

**DOI:** 10.3389/ijph.2025.1607816

**Published:** 2025-04-23

**Authors:** Solomon Mekonnen Abebe, Mikyas Abera, Ansha Nega, Zemichael Gizaw, Mulugeta Bayisa, Solomon Fasika Demissie, Molalign Belay, Abel Fekadu, Wondwossen Wakene, Getachew Azeze Eriku

**Affiliations:** ^1^ Institute of Public Health, University of Gondar, Gondar, Ethiopia; ^2^ Department of Sociology, University of Gondar, Gondar, Ethiopia; ^3^ School of Public Health, Addis Ababa University, Addis Ababa, Ethiopia; ^4^ Department of Physiotherapy, School of Medicine, College of Medicine and Health Sciences, University of Gondar, Gondar, Ethiopia; ^5^ Department of Epidemiology and Biostatistics, University of Gondar, Gondar, Ethiopia; ^6^ School of Law, University of Gondar, Gondar, Ethiopia

**Keywords:** disability, People with disbailities, DHSS, household survey, Northwestern Ethiopia, severe disability

## Abstract

**Objectives:**

To assess the severity, prevalence and reasons for disability in Northwestern Ethiopia.

**Method:**

A community-based cross-sectional study design among 17,000 households in 13 Kebeles of Dabat district. The modified 12-item World Health Organization’s Disability Assessment Schedule (WHODAS 2.0) and 7-item WHO Domains of Functioning and Health was used to collect survey data.

**Results:**

The overall prevalence of severe disability was 9.04%. This prevalence increased with age. Visual impairments were the most commonly reported type of disabilities. In 83% of the study participants, the causes of disability were modifiable, such as illness (36.93%), injury (17.81%), and congenital (10.86%). The elderly, those unable to read and write, the single and the separated were significantly associated with severe disabilities.

**Conclusion:**

This study found severe disability is highly prevalent in Dabat district. Visual impairments were the most common reported types of disabilities, followed by mobility and hearing difficulties. Most individuals with disabilities had not completed high school, and employment opportunities were limited. Disability could be prevented through early screening and timely treatment, as many of the risk factors are modifiable.

## Introduction

Disabilities can be temporary or permanent and encompass various types and risk factors [1]. Until the 1970s, the terms impairment and disability were used intrerchangeably. However, the disability movement introduced a social model distinguishes between the two concepts: impairment refers to a condition affecting the body or mind, whereas disability results from the interaction between individuals with impairments and societal barriers [2]. This distinction has significantly advanced the rights of persons with disabilities (PwDs) [3]. However, some scholars, such as Hughes and Paterson (1997), argue that this social model overlooks the rich personal experiences of impairment [4]. They proposed reintegrating bodily experiences into cultural and symbolic spaces while appreciating individualized representations of pain and social oppression.

Disability is often conceptualized as a dynamic interaction between health conditions and contextual factors [2]. This study adopts a comprehensive definition that includes physical, mental and intellectutal imapirments affecting social functionality, opportunities, and autonomy. The WHO and the World Bank (WB) estimate that approximately 15% of the world’s population lives with some form of disability, with 80% residing in low-and middle-income countries [1, 5, 6]. The prevalence of disability is increasing due to population growth, man-made and natural disasters, war, accidents, and aging.

Evidence on disability prevalence in Ethiopia is fragmented, inconsistent, and sometimes contradictory or misleading. For example, the 2007 Population and Housing Census reported that approximately 1.2% of the population had some form of disability [7]. Similary, a study at the Dabat HDSS site found a low prevalence of 1.82%. In contrast, studies in Gondar and Bahir Dar cities reported significantly higher rates. In Gondar, 34.5% of participants had limitations in basic activities of daily living (BADL) and 54.4% had limitations in instrumental actvities of daily living (IADL) [8]. The second study in Bahir Dar city reported a functional disability prevalence of 29.6% [9]. However, these findings may not be directly comparable due to differences in disability definitions and study populations. The studies in Gondar and Bahir Dar focused on functional disbaility among older adults, whereas the Dabat HDSS study assessed disability prevalence in the general population [10]. Futhermore, underreporting due to stigma and negative community attitudes towards disability is common in Ethiopia [11]. Institutional and cultural factors, such as the cost of treatment and social stigma, contribute to significant underreporting [12]. Despite variations in reported disability prevalence, mobility, visual, and hearing impairments remain the most prevalent types of disabilities in Ethiopia [13, 14].

Reliable disability data are essential for informed policymaking, planning, and programming for inclusive and sustainable development. Understanding population functioning and the need for social and rehabilitation services guides interventions that promote equal access to education and healthcare for PwDs. National and global actors rely disability statistics to ensure the effective inclusion of PwDs in development initiatives [15–17].

PwDs experience disability differently depends on various factors, including gender, age, educational level, employment status, economic level, and geographical location [15]. In Ethiopia, about 46% of PwDs are women, who face additional challenges due to patriarchy and disability-related stigma [18]. Children with disabilities are less likely to attend school or access to healthcare services, increasing their vulnerability to poverty and poor health, which lowers their quality of life [17, 18]. Due to these challenges, PwDs remain one of the most disadvantaged segment of society [19].

Previous studies in Ethiopia have focused on the prevalence of general disability and functional limtitations, primarily using census data or functional assessments of older adults. However, there is limited evidence on the prevalence, types, and causes for severe disability among PwDs in Northwestern Ethiopia. Therefore, this study aims to fill this knowledge gap by providing a comprehensive assessment based on data from the Dabat HDSS.

## Methods

### Study Area

This study aimed to assess the prevalence, types and primary reasons of severe disability in the Dabat district, Northwestern Ethiopia, where the HDSS collects longitudinal data. Dabat town, the administrative center of Dabat district, is located 60KMs Northwest of Gondar city. The district covers an area of 1,199.15 km^2^ and has an estimated population of 168,331, with an equal distribution of males and females (CSA 2013). It is organized into 5 urban and 27 rural Kebeles, the smallest administrative unit in Ethiopia, with altitudes ranging between 1,000 and 3,000 m above sea level (Dabat Rural Project Statistics, 2015). Dabat district was purposively selected as a surveillance site of the University of Gondar due to its diverse climatic conditions, which include Dega (highland and cold), Woina Dega (midland and temperature), and Kolla (lowland and hot). This was based on the assumption that morbidity and mortality rates would be vary across these three different zones. Consequently, this research is conducted at the University of Gondar’s research center in Dabat, which justifying the selection the study area.

**FIGURE 1:**
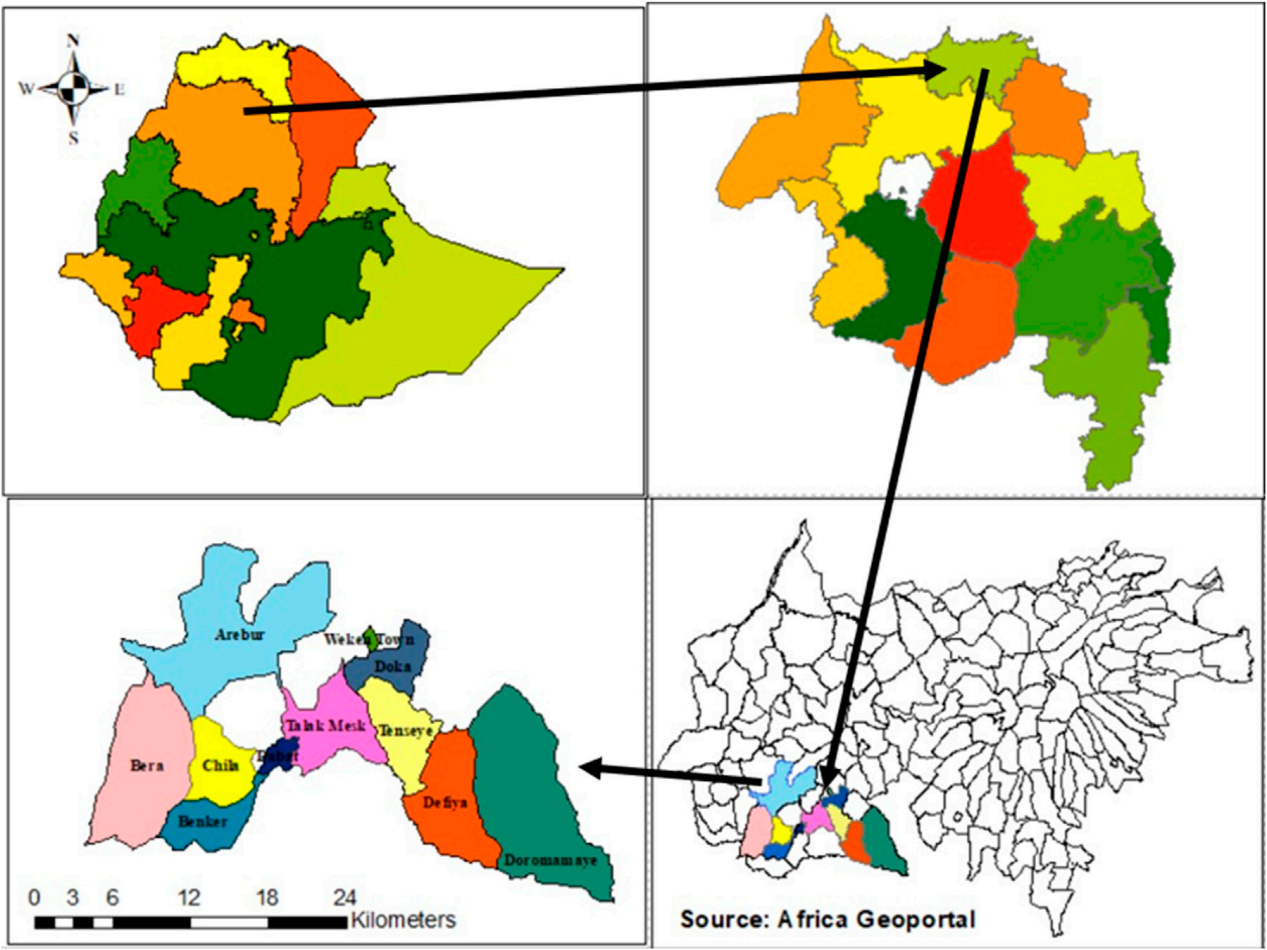
Map of the study areas in Dabat district, Gondar, Ethiopia, 2018.

The district has 29 health posts, 3 health stations, and 2 health centers. The UoG established the HDSS in 1996 to collect demographic, social and health data in 13 Kebeles (9 rural and 4 urban) out of the district’s total of 32 kebleles. According to the 2016 Re-census Baseline Survey, there are 17, 000 households with approximately 72,000 inhabitants (Figure 1).

### Study Design and Period

A community-based cross-sectional design was conducted between January to June 2018. The study aimed to assess the prevalence, types and reasons for severe disability in Dabat district. Data were collected by enumerators and supervisors who visited all households in the HDSS catchment area. The survey covered all 17,000 households across the 13 Kebele with in the HDSS site, using household numbers as a sampling frame. This survey was part of an add-on cross-sectional study launched in 2016 to address gaps in the HDSS data regarding the prevalence, types and reasons for disability. Although the add-on survey was administered separately from the HDSS, the same personnel were employed as enumerators to ensure consistency. The main aim was to generate more comprehensive disability related evidence.

### Study Population

This study included members and permanent residents of 17,000 households in 13 *Kebeles* of Dabat district.

### Sample Size Determination and Sampling Procedures

The study was conducted at the household level across the Dabat district, which includes 13 Kebeles that are already designed as the HDSS site of the University of Gondar. All 17,000 households within the HDSS site were eligible for inclusion in the survey. As this was a census-type survey, the entire household was included in the study.

The household lead, either the mother or father, was primarily a self-reported informant. This approach ensured insights into household dynamics and decision-making processes. If the household head was unavailable during data collection, one adult family member (aged 18 or above) was selected using a simple random sampling method. Individuals who had resided for less than 6 months were excluded, in accordance with the CSA definition of ‘household members at the time of data collection.

### Data Collection Tools and Procedures

The survey employed a semi-structured, pre-tested questionnaire adapted from WHODAS-2.0 [20] and the International Classification of Functioning, Disability, and Helath: children and youth (ICF-CY) [21] to assess the health and disability status of PwDs. WHODAS 2.0 was particularly used for PwDS aged 18 and above. The survey questionnaire specifically adapted items from WHODAS-2.0 that measure the six domains of disability: learning and intellectual, mobility, self-care, getting along, life activities and participation.

Thirty-three trained and experienced enumerators, employed at the HDSS site by the University of Gondar Research Center, were overseen by nine supervisors. They interviewed household heads to collect data on prevalence, types and reasons for disability among all household members. While this study collected data on the same population as the HDSS, it focused on disability using an adapted measurement tool from WHODAS 2.0 and the ICF-CY.

The questionnaire was initially designed in English and then translated to Amharic, the official language of Ethiopia, which is spoken by residents of the Dabat district. The adaptation and validation process involved piloting the questionnaire in a Kebele that was not part of the HDSS site to assess the relevance and appropriateness of its wording and item sequencing.

The pilot study included all enumerators and supervisors, who underwent 5 days of training on research methods, interviewing techniques, and the questionnaire. Researchers also participated in the process, later engaging in critical reflection and integrating the piloting results to refine the questionnaire, ensuring that the wording, sequencing, and content of the items were as relevant and appropriate as possible. Furthermore, the research team drew on their research and programming experiences in disability, community-based rehabilitation and inclusion particularly in the context of Dabat district, to ensure the cultural appropriateness and scientific validity of the tool.

Enumerators, after contacting household heads and informing them of the study`s purpose, asked them to provide information on the selected individual in the household with regarding impairment and/or disability by reading out a list of possible conditions, including hearing loss or total deafness, visual impairment or blindness, speech impairment, loss of senses or limbs, paralysis, diagnoses insanity, etc. Due to stereotypes and stigma attached to disability, enumerators asked household heads to identify if the selected individual have a disability. Enumerators then applied items from WHODAS 2.0 or ICF-CY disability information. The information on disability gathered through the community survey did not necessarily account for mild or minor impairments that household heads were either unaware of or consdeired insignificant. Consequently, the data collected and reported in this study were focused on complete or severe impairments sustained by household members.

### Data Analysis

Data were entered into the Household Registration System (v-2.1) and analyzed using STATA (v.12) software. During data cleaning and organization, unclear or incomplete items were returned to the study site for further clarification and completion. Descriptive statistics including means, percentages, and standard deviations, were used to summarize the characteristics of the study population. Tables and figures were used to present aggregated and disaggregated data, as appropriate.

Binary logistic regression was fitted to assess factors associated with the prevalence of disability. First, univariable analysis was carried out, and variables with p-values of <0.2 were included in the multivariable analysis to control for confounding factors. Results were considered statistically significant at p-value ≤0.05. The crude odds ratio (COR) and adjusted odds ratios (AOR) with 95% Confidence Interval (CI) were used to determine the association between independent variables (age, sex, place of residence, religious, education, occupation,and marital status) and severe disability using multivariable logistic regression analysis.

## Results

### Respondents’ Socio-Demographic Characteristics Among PwDs in Dabat District

Out of 17,000 surveyed households, 50.89% of participants were female, and 75.3% lived in rural areas. The socio-demographic characteristics of the survey indicate that 30.78% of participants were unable to read and write, while 27.13% were under 14 years of age. Among PwDs, 71.8% of females and 52.9% of males could not read or write. The socio-demographic characteristics of the study population (Table 1).

**TABLE 1:** Socio-demographic characteristics among persons with disabilities in Dabat district, Gondar, Ethiopia (n = 17,000), 2018.

Variables	Category	Percentage
Age (in years)	0–14 Years	30.8
	15–24 Years	24.3
	25–34 Years	15.4
	35–44 years	10.8
	45–54 years	7.66
	55–64 years	5.5
	65 and above years	5.54
Sex	Male	50.78
	Female	48.98
Marital status	Under age ˂10 years	36.8
	Married	30.66
	Single	20.2
	Divorced	3.2
	Widowed	2.58
	Separated	0.80
	Unknown	5.75
Educational attainment	Under age (˂7 years)	27.13
	Unable to read and write	30.78
	Able to read and write	6.15
	Grade 1 to 3	11.37
	Grade 4 to 6	6.27
	Grade 7 to 8	3.24
	Grade 9 to 10	4.32
	Grade 11 to 12	1.62
	Diploma and above	1.42
	Unknown	7.70
Place of residence	Urban	24.7
	Rural	75.3
Religion	Orthodox Christianity	96.71
	Islam	3.27
	Catholic/Protestant	0.02

**FIGURE 2:**
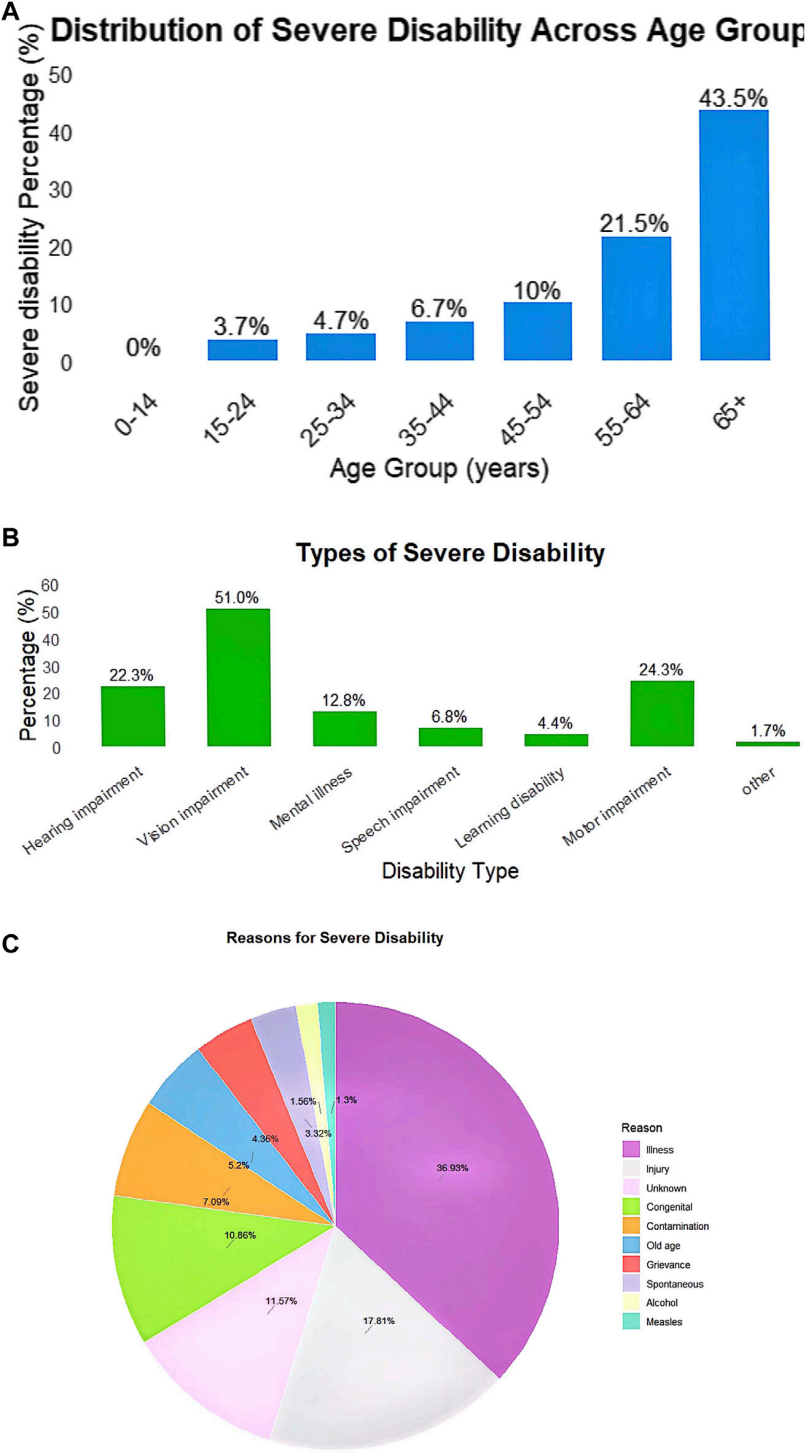
**(A)** Distribution of disability across different age groups among persons with disabilities in Dabat district, Gondar, Ethiopia, 2018. **(B)** Types of severe disability among persons with disabilities in Dabat district, Gondar, Ethiopia, 2018. **(C)** Reasons for severe disability among persons with disabilities in Dabat district, Gondar, Ethiopia, 2018.

The overall prevalence of severe disability in Dabat district was 9.04% among the surveyed households. There was higher among the older age groups, reaching its highest level among individuals aged 65 years and above is 43.5% (Figure 2A). The prevalence was slightly higher among females (9.71%) than males (8.42%). Prevalence was lower among individuals with higher educational level.

The data highlights a significant disparity in education among PwDs. A total of 18.6% had no formal education, which correlated with higher disability rates. In contrast, individuals with higher education levels (Grade 11/12) had a much lower disability rate (3.99%). Furthermore, the prevalence of disability was highest among those who were separated (40.1%) and divorced (19.1%). The difference in disability prevalence between urban (8.64%) and rural (9.17) 15%) Kebeles in the Dabat district was marginal (Table 2).

**TABLE 2:** The factors associated with severe disability among persons with disabilities in Dabat district, Gondar, Ethiopia, 2018 (n = 17,000).

Variables	Categories	Severe disability		COR (95%CI)	AOR (95% CI)	p-value
		Yes	No			
Age	<65	1,127 (7.0)	14,931 (93.0)	1.0	1.0	
	≥65	410 (43.5)	532	1.04 [1.037, 1.042]	1.04 [1.03,1.04]*	<0.001
Gender	Male	700 (8.42)	7,917 (91.58)	1.00	1.00	
	Female	837 (9.71)	7,476 (90.29)	2.49 [0.23, 27.5]	1.06 [0.94, 1.18]	0.362
Educational level	Under age (˂7 years)	153 (3.32)	4,459 (96.68)	1.00	1.00	
	Unable to read and write	971 (18.56)	4,261 (81.44)	5.74 [4.84, 6.82]	1.57 [1.15, 2.13]	0.004
	Able to read and write	130 (12.44)	915 (81.56)	3.82 [3.02, 4.84]	1.10 [0.77, 1.57]	0.588
	Grade 1 to 3	88 (4.55)	1845 (85.45)	1.37 [1.06, 1.79]	0.64 [0.45, 0.90]	0.01
	Grade 4 to 6	57 (5.35)	1,009 (94.65)	1.61 [1.19, 2.19]	0.58 [0.39, 0.87]	0.009
	Grade 7 to 8	36 (6.55)	515 (93.45)	1.98 [1.38, 2.86]	0.69 [0.44, 1.09]	0.112
	Grade 9 to 10	41 (5.58)	694 (94.42)	1.69 [1.19, 2.39]	0.60 [0.39,0.94]*	0.025
	Grade 11 to 12	11 (3.99)	265 (96.01)	1.21 [0.65, 2.23]	0.35 [0.18, 0.69]*	0.002
	Diploma and above	15 (6.22)	226 (93.78)	1.88 [1.10, 3.21]	0.54 [0.29, 0.99]*	0.047
	Unknown	35 (2.67)	1,274 (97.33)	0.81 [0.56, 1.16]	0.44 [0.23, 0.84]*	0.012
Marital Status	Under age ˂10 years	209 (3.34)	6,047 (97.66)	1.00	1.00	
	Married	664 (12.74)	4,548 (87.26)	3.89 [3.32, 4.55]	0.79 [0.58, 1.07]	0.136
	Single	287 (8.35)	3,148 (81.65)	2.52 [2.11, 3.02]	1.86 [1.39, 2.48]*	<0.001
	Divorced	104 (19.1)	440 (80.9)	5.89 [4.64, 7.49]	1.02 [0.71, 1.46]	0.931
	Widowed	176 (40.09)	263 (59.91)	12.9 [10.48, 15.8]	1.17 [0.81, 1.69]	0.396
	Separated	63 (40.1)	73 (59.9)	14 [10.6, 19.12]	4.15 [2.78, 6.19]*	<0.001
	Unknown	0 (0.00)	978 (100%)	0.77 [0.51, 1.17]	2.88 [1.52, 5.49]*	0.001
Place of Residence	Urban	363 (8.64)	3,836 (91.36)	1.00	1.00	
	Rural	1,174 (9.17)	11,627 (90.87)	1.26 [1.10, 1.45]	0.98 [0.86, 1.12]	0.8

8% of PwDs had more than one type of disability. Regarding the type of disabilities, 51% had visual impairment, 24.3% had mobility impairment, and 22.3% experienced hearing difficulties (Figure 2B).

The survey identified the most frequent reasons for disability as illness (36.93%), followed by accidents (17.81%), unknown reasons (11.57%), congenital conditions (10.86%), and ageing (5.2%). Notably, more than 83% of the reported immediate reasons for disability could have been prevented from leading functional limitations if they had been identified and treated early (Figure 2C).

### Factors Associated With Severe Disability Among PwDs in Dabat District

The findings indicate that increasing age [AOR = 1.04; 95% CI: 1.03, 1.04], educational attainment (unable to read and write [AOR = 1.15; 95% CI: 1.57, 2.13], grade 1–3 [AOR = 0.64; 95% CI: 0.45, 0.90], grade 4–6 [AOR = 0.56; 95% CI: 0.39, 0.87], grade 9-10 [AOR = 0.60; 95% CI: 0.39, 0.94], grade 11–12 [AOR = 0.35; 95% CI: 0.18, 0.69], and diploma and above [AOR = 0.44; 95% CI: 0.23, 0.84]), and marital status; being single [AOR: 1.39; 95% CI: 185, 2.47] and separated [AOR: 2.78; 95% CI: 4.14, 6.19] were significantly associated with severe disability in our study population (Table 2).

## Discussion

This study found that, although the overall prevalence of disability was 9.04%, it significantly affected older individuals in the community. The proportion of individuals with disability at the household level was higher compared to previous studies in the study area. While concerns about reliability, representativeness or timeliness exist, CSA previous studies reported disability prevalence of 1.82% [22] and 1.09% [23] in Dabat district, which were significantly lower than the findings of this study. The obsereved difference could be due to the methodological variations. The previous cross-sectional study conducted in the same area, at Dabat HDSS site, used the entire population residing in selected kebeles as the denominator, whereas our study used households as the denominator. This difference in methodology likely contributed to the discrepancy in disability prevalence. Additionaly, the previous study utilized re-census data, which may have understimated or overlooked cases. However, despite the 9% prevalence found in this study, it remains significantly lower than 17.6% national disability prevalence rate reported by WHO and WB in 2011 [10, 16].

There are several possible explanations for the discripancies of finding in disability prevalence across different sources. First, the WHO conceptualizes disability broadly, incorporating various factors and types, such as chronic illnesses as diabetes, as well as moderate and minor impairments. However, in resource-limited settings, where rehabilitation services are sacre, narrower operationalization of disability may have been adopted.

The pragmatic conceptualization of disability may influence policy formulation and social work practices. Second, this study relied on responses provided by household heads to estimate the prevalence of disability. The quality of data could have been affected by respondents’ lack of knowledge regarding their own or household members’ disabilities, as well as negative attitudes toward disability and PwDs. Consequently, the stigma surrounding disability in the district and respondents’ inability to identify all types and degrees of disability may have led to underreporting of disability.

Regarding the type of disability, this study reported findings similar to a previous study conducted by the same research team in Dabat district [22]. Both studies found visual impairment as the most common disability. However, while the current study found mobility and hearing impairments to be the second and third most prevalent disabilities, their relative proportions were reversed 3 years earlier [22].

A small fraction of PwDs attended formal education, and as their educational level advanced, their progression to higher grades decreased significantly. This finding aligns with the Handicap International report, which reported that only 3% of the 2.4 to 4.8 million school-age children with disabilities (CwDs) attend school. Several factors contribute to low school attendance and participation among CWDs, including stigma, inaccessible schools, rigid teaching practices, a lack of trained teachers who can accommodate students with special needs, and insufficient adaptive learning resources [24]. A study in South Africa also documented how PwDs and their families experience stigma and social exclusion, affecting their personal development and community participation. Other studies have similarly reported that CwDs are less likely to attend school, access healthcare, and are more vulnerable to poverty, which can significantly reduce in their quality of life [25–27].

This study found that the majority of reasons for disability reported were modifiable such as illnesses, and injuries indicating that the prevention strategies could be effective. Studies have similarly documented that most disabilities in Africa result from preventable illness, injury, and accidents [14, 28]. In Ethiopia, diseases such as measles, poliomyelitis, trachoma as well as accidents, contribute significantly to disability. However, early identifictaion or timely treatment could mitigate functional and activity limitations. While this does not imply that all disabilities can be entirely prevented, early management of conditions such as trachoma, and measles could reduce the severity of impairment and enhance functionality. This scenario is not unique to Ethiopia; for instance, a study on nine Latin American countries found that 80% of visual impairments were avoidable with early detection and treatment [29–31].

Moreover, most disabilities and their causes are linked to poverty and restricted access to basic services. As reported in the study, most PwDs both in urban and rural areas were economically disadvantaged, with a significant majority engaged in small-scale farming under strained livelihoods. Additionally, inadequate healthcare services, poor health literacy, and ineffective health-seeking behaviors contribute to the disability burden, as observed in Dabat district. Similarly, disability is associated with serious negative health outcomes, and long-term care for PwDs in resource-limited settings remains a major public health issue. Disability is particularly problematic in developing countries, where preventive approaches and healthservices are either inadequate or scarce [5, 32]. Furthermore, stigma and stereotypes limit educational and employment opportunities for PwDs, leading to dependency and social exclusion [33]. Women with disabilities face an additional burden experiencing both gender-based discrimination and economic challenges. Disability and poverty exacerbate their socioeconomic conditions and overall quality of life [34].

As a strength, this study explored the prevalence, types, and reasons for severe disability in a resource-limited setting, in Dabat district, Northwestern Ethiopia. Although studies have been conducted in Ethiopia, particualry in this area, our study assessed severe disability using mulitple questions rather than a single yes/no as reponse, as was done in previous studies. This approach provided a more detailed understanding of severeity of disability, types and its associated factors. Furthermore, this study highlighted the lack of community and governmental attention towards disability and the empowerment of PwDs in developing countries such as Ethiopia.

A key limitation of this study is its reliance on self-reporting data rather than clinical diagnoses, which may have affected the accuracy of the findings. Participants may have lacked knowledge about the nature and degree of disability potentially leading to underreporting. Whenever new cases of disability were identified during data collection, they were referred to or linked with local Community-Based Rehabilitation (CBR) fieldworkers.

Another limitations arises from relying on household heads to report the presence of PwDs in their households and focusing on severe or extreme functional limitations rather than the full spectrum disabilities. To mitigate first limitation, the researchers provided a list of possible impairments and disabilities to help household heads frame their responses. However, due to the large sample size (17,000 households), conducting comprehensive disability screening was not feasible. Consequently, the reported disability prevalence in the district was significantly lower than the WHO estimate for low- and middle-income countries. It is important to consider that household heads primalrily reported visible and severe impairments, which may have led to underreporting of less visible or milder disabilities.

In conclusion, this study found the proportion of self-reported severe disability was high. Visual amd mobility imapirments were the most frequently reported types of disability. Most reported reasons for disability were modifiable, incuidng illness and injury. However, structural factors such as poverty, aging, inaccessibility of health services require programmatic interventions at national, regional and local levels. The prevalence of severe disability increased with age. Additionally, individuals with lower levels of education were more likely to report severe disabilities, refinfcing the importance of acces to education for individuals with disabilities.

Further qualitative research is recommended to better understand the lived experiences of PwDs and the societal barriers they faced. Futhermore, quantitative research using clinical evaluation is recomemeded to provide a clear picture of the prevalence, severity and determinant of disability.

## Data Availability

Since the data presented in this report are part of the large HDSS survey, we must adhere to the University of Gondar’s data sharing policey. Nonetheless, we have included all relevant information in the tables and figures presented (No additional data are available).

## Abbreviations

ANRS, Amhara National Regional State; CBR, Community-based Rehabilitation; CSA, Central Statistical Agency; CwDs, Children with Disabilities; DHSS, Demographic Health Surveillance System; ICF-CY, International Classification of Functioning, Health and Disability: children and youth; ICF-CY, Health and Disability, Children & Youth version; NGZ, North Gondar Zone; PwDs, Persons with Disabilities; WB, World Bank; WHO, The World Health Organization; WHODAS 2.0, World Health Organization Disability Assessment Schedule.
